# Directed Evolution of *Saccharomyces cerevisiae* for Increased Selenium Accumulation

**DOI:** 10.3390/microorganisms6030081

**Published:** 2018-08-06

**Authors:** Masafumi Yoshinaga, Stephanie How, Damien Blanco, Ian S. Murdoch, Matteo Grudny, Samantha L. Powers, Nelson Molina, Barry P. Rosen, Aaron Z. Welch

**Affiliations:** 1 Department of Cellular Biology and Pharmacology, Herbert Wertheim College of Medicine, Florida International University, Miami, FL 33199, USA; myoshina@fiu.edu (M.Y.); brosen@fiu.edu (B.P.R.); 2 Biomolecular Sciences Institute, Florida International University, Miami, FL 33199, USA; show001@fiu.edu (S.H.); damien.p.blanco@gmail.com (D.B.); imurd001@fiu.edu (I.S.M.); mgrudny@fiu.edu (M.G.); spowers@fiu.edu (S.L.P.); nmoli015@fiu.edu (N.M.)

**Keywords:** yeasts, resistance, fermentation biotechnology, Fungi, metabolic processes, selenium

## Abstract

Selenium-enriched yeast (selenium yeast) are one of the most popular sources of selenium supplementation used in the agriculture and human nutritional supplements industries. To enhance the production efficiency of selenium yeast, we sought to develop a method to identify, and ultimately select for, strains of yeast with enhanced selenium accumulation capabilities. Selenite resistance of four genetically diverse strains of *Saccharomyces cerevisiae* was assayed in various conditions, including varying carbon sources, nitrogen sources, and phosphate amounts, and they were correlated with selenium accumulation in a commercially relevant selenium-containing growth medium. Glycerol- and selenite-containing media was used to select for six yeast isolates with enhanced selenite resistance. One isolate was found to accumulate 10-fold greater selenium (0.13 to 1.4 mg Se g^−1^ yeast) than its parental strain. Glycerol- and selenium-containing medium can be used to select for strains of yeast with enhanced selenium accumulation capability. The methods identified can lead to isolation of industrial yeast strains with enhanced selenium accumulation capabilities that can result in greater cost efficiency of selenium yeast production. Additionally, the selection method does not involve the construction of transgenic yeast, and thus produces yeasts suitable for use in human food and nutrient supplements.

## 1. Introduction

Selenium is an essential element for humans and animals. It is naturally found in its inorganic form in the crust of the earth, dispersed throughout the world, though it is more concentrated in some regions than others [1]. Selenium normally enters the food chain as plants absorb inorganic selenium, in the form of the oxidized species selenite (Se(IV)) or selenate (Se(VI)), from soil, and convert this into organic, carbon-bound selenium. However, the amount obtained naturally is often insufficient for the optimal growth and development of animals; therefore, to meet optimal levels in animals, organic selenium supplements are often added to animal feed [2]. Selenium supplements may also be beneficial for humans by reducing cancer incidence, although this is controversial [3]. One of the most popular sources of selenium supplements is selenium-enriched *Saccharomyces cerevisiae* (selenoyeast). As such, studies have focused on understanding the uptake mechanisms, metabolism, and toxicity of selenite in yeast [4,5,6]. There are currently several commercially available selenium-enriched yeast strains such as Lalmin SE2000, Selenoexcell, and Sel-Plex. To create selenium-enriched yeast, cells are grown in liquid medium with a species of inorganic selenium present, which is then taken up and converted to an organic species that is more easily assimilated by animals. Common forms of organic selenium include selenodiglutathione, and the amino acids selenomethionine and selenocysteine. The exact protocol used by selenium-enriched yeast manufacturing companies is proprietary, although several academic papers have suggested optimized protocols for selenium accumulation in yeast [6,7]. Selenium yeast are sold in the form of dry powder, and contain anywhere from 1–2 mg selenium per gram dry weight. In the laboratory, researchers have achieved >98% replacement of methionine by selenomethionine, and up to 3 mg total selenium per g dry weight [4,8,9]. This is significantly greater than the amounts of selenium in commercial yeast. However the theoretical limit of selenium incorporation is around 6 mg g^−1^ dry weight [4]. These observations suggest there is room for enhancement of selenium content of commercial selenium yeast. One reason for the disparity between selenium content in commercial and laboratory strains of yeast is due to a precise, and costly, manipulation of laboratory growth medium into which the selenite and yeast are inoculated. These changes to medium are likely to be too costly to reproduce on a commercial scale.

Besides manipulation of the medium, another approach to increase commercial yeast’s selenium accumulation is through genetic enhancement. One approach would be to screen for increased selenium resistance, as measured by faster growth in medium containing selenite than wild-type cells, which may result in increased selenium accumulation. There are reports of mutations leading to increased selenium resistance. For example, overexpression of a sulfite-efflux gene SSU1, deletion of low-affinity phosphate transporters, or deletion of a proton-lactate symporter JEN1, results in increased selenite resistance [8,9,10]. The likely mechanism of selenite resistance due to these mutations are through either increased efflux of selenite, or deletion of a selenite transporter, respectively, and thus would not result in increased selenium accumulation. While these mutants are more resistant to selenite, they would not be more tolerant (i.e., able to grow with increased intracellular selenite). Two instances of possible selenite tolerance are from overexpression of the glutathione reductase gene GLR1, or deletion of YCF1, a selenodiglutathione vacuolar transporter [10]. The intracellular selenite levels or selenium accumulation in these mutants have not been verified. The mechanism of action of these mutations appears to be through the reduction of the lethal oxidative stress experienced by the cell due to high doses of selenite. Once selenite enters the cell, to mitigate its toxicity, it must be transformed to less toxic organic forms such as selenomethionine or selenocysteine. This requires a metabolic state that is capable of producing significant reducing equivalents in the form of glutathione (GSH), NADH, or NADPH. The redox state of the cell depends on several factors, some of which are the carbon, nitrogen, and phosphate sources that it uses for growth.

The putative selenite-tolerant mutants mentioned above would not be suitable for commercial use because they are genetically modified (i.e., constructed using recombinant DNA techniques). Many consumers remain fearful of genetically modified organisms (GMOs), and in some countries these organisms are banned from the food supply. Thus there is a need for a natural selection or screening method to increase the selenite tolerance of yeast strains. The objective of this study was to analyze various nutrition sources for their suitability to select for increased selenium accumulation in optimized selenium accumulation medium (OSAM). Hence we did not seek to identify new media formulations that led to increased selenium accumulation, but only to identify media conditions in which selenite resistance is proportional to selenium accumulation in another medium. This could be exemplified by a medium in which low selenium-accumulating strains would show little resistance to selenite, while high selenium-accumulating strains would be resistant. This medium could then be used to identify strains that are resistant to selenite, and we would predict that they would be able to accumulate high amounts of selenium under optimized selenium accumulation conditions.

The factors that determine selenium accumulation or selenite resistance can be influenced by the rates of selenite uptake or, reduction, adsorption to the cell wall and toxicity. All of these factors are important to different degrees in determining either selenium accumulation or resistance, and are modulated by the medium. Our goal was to examine various media conditions to determine which conditions cause the greatest overlap in the factors influencing selenite resistance and accumulation. We measured the degree of overlap as a correlation between yeast strain-specific selenite resistance and selenium accumulation in OSAM. We found one medium that produced a significant correlation between selenium resistance and selenium accumulation in OSAM, and successfully used this medium to select for a strain with increased selenium accumulation.

## 2. Materials and Methods

### 2.1. Strain and Plasmid Construction

The strains used in this study are listed in Table 1. Strain AW024 was created by sporulating EC1118 and obtaining a heterothallic haploid strain. This strain was grown in a culture and an isolate that was 5-fluoroorotic acid resistant was selected, and found to require uracil. EC1118 is a yeast strain commonly used for wine fermentation [1]. The Lalmin strain was an isolate recovered from a dry commercial selenium yeast powder of Lalmin^®^ SE2000 (Lallemand, Milwaukee, WI, USA). Yeast strain AW063 is a haploid derivative of laboratory strain S288c and 1893-II-6C. AW106 is an isolate resulting from the plating of S288c by plating to rich medium with 20 g L^−1^ glucose and 20 mM selenite.

### 2.2. Media and Growth Conditions

Synthetic complete (SC) medium contained 3.6 g L^−1^ yeast nitrogen base without amino acids or ammonium sulfate, 12.5 g L^−1^ ammonium sulfate, 20 g L^−1^ glucose, and 20 mg L^−1^ of amino and nucleic acids histidine, uracil, leucine, lysine, phenylalanine, adenine, arginine, methionine, tyrosine, and tryptophan. Optimized selenium accumulation medium consisted of 0.5 g L^−1^ Yeast nitrogen base (YNB) with 10 g L^−1^ urea with 80 g L^−1^ molasses with 200 µmol L^−1^ sodium selenite. During the measurement of growth rates in varying carbon sources, the medium consisted of either 30 g L^−1^ ethanol, 30 g L^−1^ glycerol, 20 g L^−1^ galactose, 18 g L^−1^ lactate, or 20 g L^−1^ glucose in 0.5 g L^−1^ YNB + 10 g L^−1^ urea + 4 mg L^−1^ amino and nucleic acids, with 0 or 3 mmol L^−1^ selenite. During the measurement of growth in various nitrogen sources, the medium consisted of either 55 g L^−1^ monosodium glutamate, 22 g L^−1^ ammonium sulfate, 6.5 g L^−1^ allantoin, or 10 g L^−1^ urea in 1.8 g L^−1^ YNB without ammonium sulfate or amino acids with 80 g L^−1^ glucose with 4 mg L^−1^ amino and nucleic acids. The amounts of the various nitrogen sources used were equal in nitrogen molarity. During the measurement of growth rate in varying phosphate concentrations, the base medium used was synthetic complete medium lacking phosphates (5.7 g L^−1^ YNB with ammonium sulfate, without phosphates (MP Biomedicals) + 10 g L^−1^ urea + 80 g L^−1^ glucose + 4 mg L^−1^ amino acids or bases), with the indicated concentrations of sodium selenite and potassium phosphate added. Rich medium is composed of 12.5 g L^−1^ yeast extract with 24.6 g L^−1^ peptone. Culture density was determined by a spectrophotometer reading of the optical density at 600 nm. UV-mutagenesis was performed in a Spectrolinker emitting light at 254 nm to a lethality of between 20% to 90%.

### 2.3. Measurement of Selenium Content

Yeast were inoculated to synthetic complete medium and allowed to grow for 16 h. Then cells were washed in deionized water, and inoculated to OSAM at an A_600nm_ of between 2 and 4. Cells were grown in a shaking water bath set to 30 °C for 3 days, after which cells were washed three times in deionized water to remove extracellular selenite. To measure dry weight, cells were dried in a centrifuge under constant vacuum, then weighed. To measure wet weight, cells were spun down in glass tubes, and the supernatant solution was removed from the yeast pellet. Wet or dry yeast samples were then digested with 500 µL 70% nitric acid and heated to a temperature of 121 °C and pressure 15 psi for 1 h. The supernatant solution was then diluted in deionized water and measured for the quantity of ^82^Se using inductively coupled plasma mass spectrometry. The amount of selenium present in the yeast was divided by the dry/wet weight of the sample to obtain the mg of selenium per gram dry/wet weight yeast.

### 2.4. Growth Rates 

Strains were freshly grown overnight in synthetic complete medium, then they were inoculated to an A_600nm_ = 0.08 in 200 µL of various media in 96-well plates with a lid on it to prevent evaporation. The A_600nm_ was measured in 96-well plates without shaking, at 30 °C every 30 min for 48 h. This technique gave the most reproducible results, as opposed to various shaking regimes. Growth curves were smoothed, by removing points that only led to a temporary increase (spike). The final absorbance was divided by the initial absorbance for each 30 min interval, then each fraction was averaged with the two times preceding and proceeding it, and the maximum average fraction of the 48 h observance period was used to calculate the doubling time. Doubling time (DT) was calculated according to the following equation:

(1) $$Af=Ai\times 2^{\frac{30\;\min}{\mathrm{DT}}}$$

where *Af* is final absorbance, *Ai* is initial absorbance. The limit of detection was defined as the average of the fastest “doubling” well not containing medium (noise), which was 230 h. Thus the doubling rate upper limit was set at 230, which equates to non-detectable growth. For measurement of the effects of carbon source and phosphate limitation growth curves were repeated at least five times. For measurement of the effects of various nitrogen sources growth curves were repeated at least six times. For measurement of growth in rich medium with glycerol, growth curves were repeated five times.

### 2.5. Wet-to-Dry Weight Conversion

Twenty samples of EC1118 wet weights were recorded, then these were dried overnight, and the corresponding dry weight was recorded. A plot of wet versus dry weight was created, and a linear correlation was performed, and an equation of *y* = 0.2412*x* was calculated that gave an *r* = 9942. Wet weights were multiplied by 0.2412 to convert to dry weight.

### 2.6. Statistical Analyses 

All statistical analyses were performed using the Microsoft Excel (Version 2016 MSO 16.0.4266.1001). Linear correlation used the function CORREL to derive a Pearson product-moment correlation coefficient (*r*-value). Using the *t*-value and the degrees of freedom a non-directional *p*-value was calculated for linear regression. For calculation of the *p*-value using the students *t*-test for selenium accumulation in AW024 compared with AW024-3, the values corresponding to selenium accumulation in at least three trials were used, and an unpaired homoscedastic, one-tailed *t*-test was conducted. For comparison of doubling times in conditions with or without selenite, Welch’s two-tailed *t*-test was used. Significance level was set at 0.05.

## 3. Results

**Figure 1:**
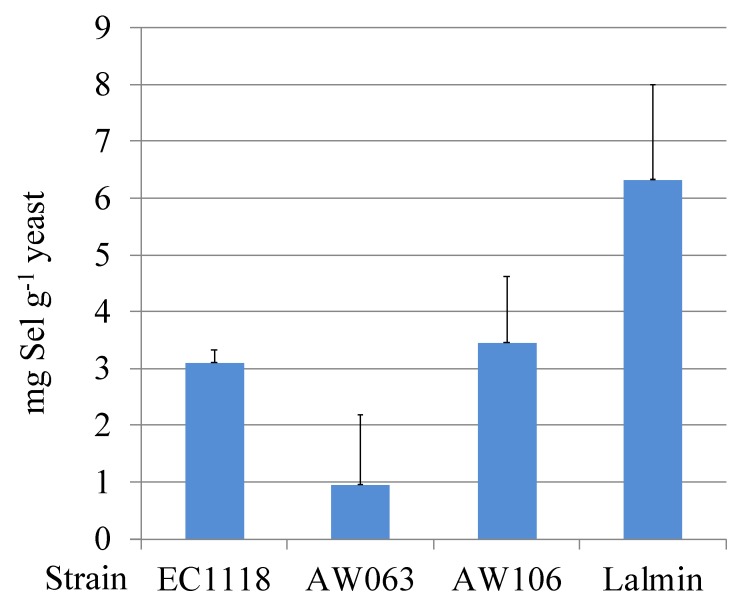
Selenium accumulation capability of selected strains. Yeast grown in optimized selenium accumulation medium (OSAM) were assayed for their selenium content per gram of yeast.

The goal of this study was to uncover any significant correlations between selenium accumulation in OSAM and selenite resistance in *Saccharomyces cerevisiae* under various culture conditions. First, we selected four diverse strains of *S. cerevisiae* to study (Table 1). To determine the selenium accumulation capability of these strains, they were grown in yeast nitrogen base, with urea as the nitrogen source, and non-sulfited molasses as the carbon source with 200 µM sodium selenite added, as previously recommended for efficient, low-cost, selenium accumulation [6]. This growth medium is referred to as optimized selenium accumulation medium (OSAM). Limited sulfur is important to encourage selenite uptake, as these elements compete with each other [4]. Cultures were incubated until stationary phase, and then total selenium content was measured using an inductively coupled plasma mass spectrometer (ICP-MS; Figure 1).

Under these growth conditions, the Lalmin strain accumulated the highest amount of selenium, at 6.3 mg Se g^−1^ dry weight. Strains AW106 and EC1118 accumulated similar amounts of selenium, at 3.5 and 3.1 mg Se g^−1^ dry weight, while AW063 accumulated the least selenium at 1 mg Se g^−1^ dry weight. Presumably, these differences in selenium accumulation reflect genetic differences in these yeast strains, as their preparation was identical.

### 3.1. Correlations of Selenite Resistance and Selenium Accumulation

**Figure 2:**
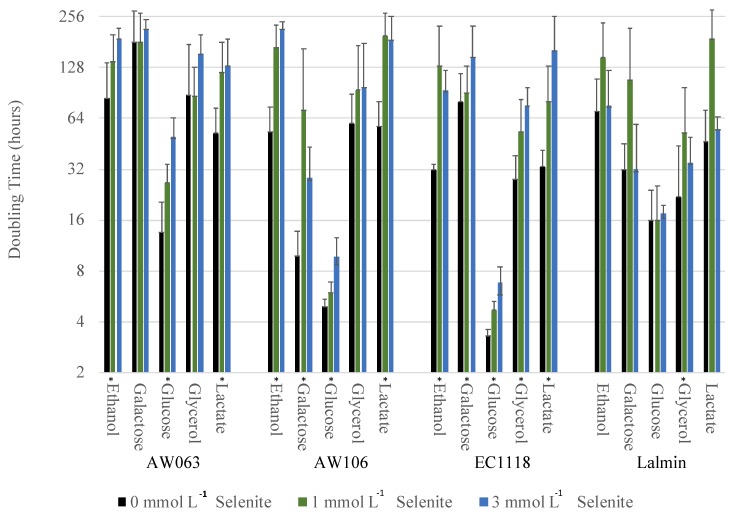
Selenite resistance dependence on carbon source. Strains were grown overnight in synthetic complete medium, then inoculated into medium containing the indicated carbon source and the indicated concentration of selenite. Growth rate was monitored for 48 h, and maximal growth rate (doubling time) was determined as described in methods. Doubling times are displayed on a logarithmic scale. Asterisk above carbon source label indicates a significant difference (*p* < 0.05) in doubling time between 0 and 3 mmol L^−1^ conditions.

Next, we determined if the amount of selenium accumulated in OSAM was correlated with resistance to selenite in other media. The first condition we investigated was growth in medium containing various carbon sources with selenite. Yeast were grown overnight in synthetic complete medium, then inoculated into either synthetic medium containing either ethanol, galactose, glucose, glycerol, or lactate with varying concentrations of selenite, and their doubling rates were measured (Figure 2).

In general, addition of selenite decreased the growth rate (increased doubling time) of the strains. However, specific deviations from this trend were noted, for example, the EC1118 and Lalmin strains showed minimal increases in doubling time due to increased selenite concentrations when grown in glucose. Strains AW063 and AW106 have mutations affecting galactose utilization (*∆gal7* and *∆lgal2*, respectively), though only AW063 exhibits an inability to utilize galactose.

To quantify the effect of selenite on growth rate for the various strains, the doubling time was plotted with selenite concentration for each strain under each carbon condition and a slope and linear regression for each condition was calculated (Table 2). A lower slope indicates greater resistance to selenite. Next, the correlation between selenite resistance slope in differing carbon media with selenium accumulation in OSAM was calculated. For each carbon source *r*-, *t*- and *p*-values were calculated (Table 2). Note that the selenium accumulation was not performed in differing carbon source conditions but only in OSAM.

Resistance to selenite while growing in ethanol, galactose, glucose, and lactate did not correlate with their selenium accumulation in OSAM. However, resistance to selenite when grown in glycerol was significantly correlated with selenium accumulation in OSAM with a *p*-value = 0.013.

**Figure 3:**
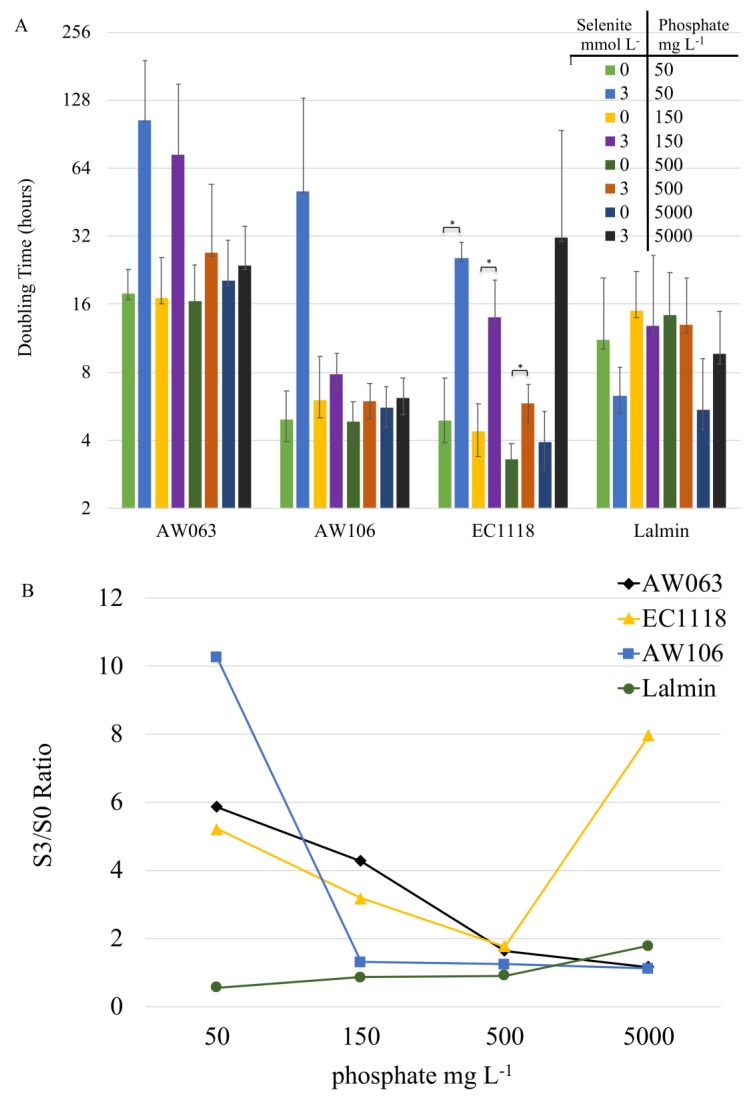
Growth rates of yeast strains in conditions of varying selenite and phosphate. Strains’ growth rate in the indicated amounts of sodium selenite or potassium phosphate was monitored for 48 h, and doubling time determined. (**A**) Doubling times are displayed on a logarithmic scale. (**B**) The S3/S0 ratio was determined by dividing the doubling time of the strain in 3 mmol L^−1^ selenite by the doubling time of the strain in 0 mmol L^−1^ selenite. Yeast strains are represented as EC1118 (▲), AW106 (■), Lalmin (●), and AW063 (♦). Bar with asterisk indicates a significant difference (*p* < 0.05) in doubling time between 0 and 3 mmol L^−1^ conditions.

Phosphate transporters, and their regulation via phosphate concentration, could be another variable that contributes to the interplay of selenite resistance and accumulation. Low phosphate conditions have been shown to upregulate low-affinity phosphate transporters, which causes an increase in selenite uptake [5]. To examine the effect of phosphate concentration on selenite resistance, strains were grown under conditions of low (50 mg L^−1^) to high (5 g L^−1^) phosphate, with and without selenite (Figure 3A). For reference, standard yeast synthetic medium contains 1 g L^−1^ phosphate. No significant difference in growth rates was observed due to varying phosphate concentrations without selenite. However, the addition of selenite decreased the growth rate in tandem with phosphate limitation in EC1118, AW106, and AW063 strains. These results are in agreement with previous observations that phosphate limitation causes selenite sensitivity [5].

To determine if a strain’s selenium accumulation was proportional to its phosphate concentration-dependent selenite resistance (S3/S0 ratio) was calculated by dividing the doubling time of strains grown in 3 mmol L^−1^ selenite by the doubling time of strains grown without selenite. The S3/S0 ratio serves as a proxy for selenite resistance, with a lower ratio indicating greater resistance. Strains exhibited distinct S3/S0 ratio trends, with some strains having greater variation in their S3/S0 values, depending upon the concentration of phosphate (Figure 3B). For AW063, EC118, and AW106, decreasing amounts of phosphate led to reduced resistance. The Lalmin strain displayed either no or decreased effect on selenite resistance due to phosphate depletion. A linear regression fit to each of S3/S0 ratio and correlation with selenium accumulation did not yield a significant correlation (*r*-value 0.92, *p*-value 0.076). Thus, while it seems that phosphate limitation does generally decrease resistance to selenite, it is not proportional to the level of selenium accumulation in OSAM.

**Figure 4:**
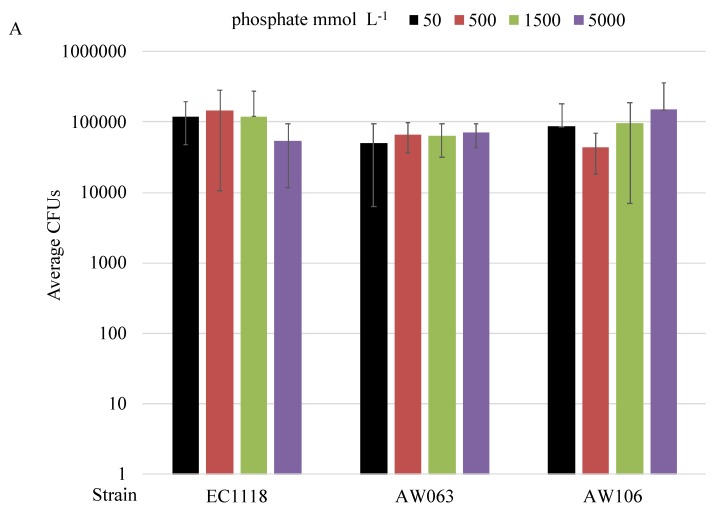

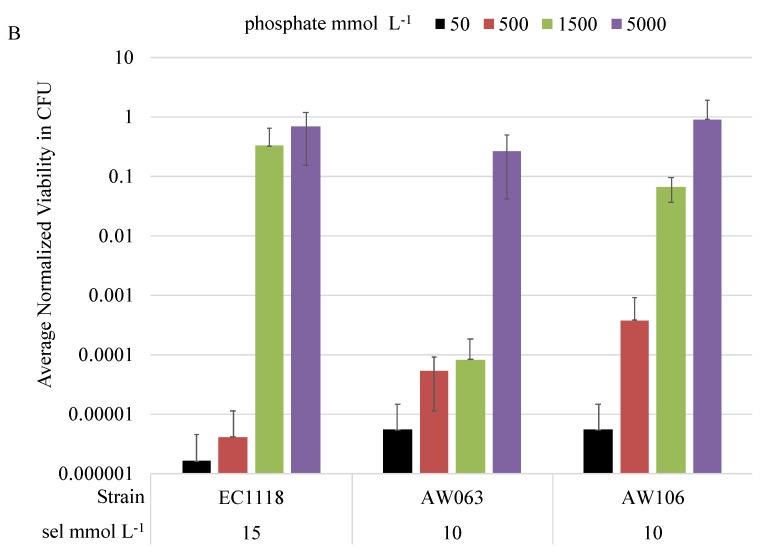
Colony forming ability of yeast strains in phosphate-limited medium with selenite. Strains were grown overnight in medium lacking phosphate, then tadpoled into medium containing the indicated amount of phosphate without selenite (**A**) or with sodium selenite (**B**). The concentration of sodium selenite added varied by strain, with EC1118, AW063, and AW106 receiving 15, 10, and 10 mmol L^−1^ sodium selenite respectively. Cultures grew for up to seven days, then colonies were counted (CFUs). The number of colonies formed in medium containing selenite was divided by the number of colonies formed in medium lacking selenite to calculate the Normalized Viability in CFU. These results were averaged over three experiments. Results shown on a logarithmic scale.

To examine additionally if phosphate-limitation affects selenite resistance, a liquid colony-forming assay, termed tadpoling, was employed [11]. In this assay, cells are serially-diluted in liquid medium in a 96-well plate that is kept stationary until colonies form, which are then quantified to determine a culture viability. To do this, cells were grown in medium lacking phosphate overnight, then they are transferred to medium containing differing amounts of phosphate with or without selenite and normalized viability was obtained by dividing the number of colonies that formed in medium with selenite by the number of colonies that formed in medium without selenite. The concentration of selenite was adjusted for each strain such that sub-lethal levels were obtained in phosphate replete conditions. The strain EC1118 was more resistant than strains AW063 and AW106 and as such, the selenite treatment was greater for this strain. The Lalmin strain was not used in this assay as it did not form colonies in liquid medium. There was no significant difference in the number of colonies formed due to varying amounts of phosphate in medium lacking selenite (Figure 4A). However, there was a difference in the number of colonies formed due to varying amounts of phosphate in medium containing selenite (Figure 4B). This trend was observed in all strains tested. These results suggest that phosphate concentration affects selenite resistance in the strains tested.

**Figure 5:**
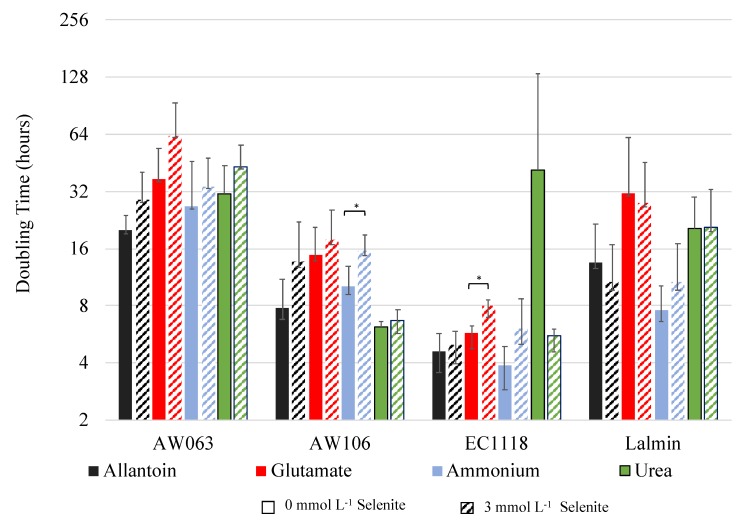
Dependence on the nitrogen source of selenium resistance. Strains were grown overnight in Synthetic complete medium then inoculated into medium containing equal nitrogen molar concentrations of the above compounds. The doubling time of strains inoculated into 3 mmol L^−1^ selenite containing medium is indicated by dotted columns. Their growth rate was monitored for 48 h, and maximal growth rate (doubling time) was determined as described in methods. Doubling times are displayed on a logarithmic scale. Bar with asterisk indicates a significant difference (*p* < 0.05) in doubling time between 0 and 3 mmol L^−1^ conditions.

Another media component that could influence selenite resistance that has not previously been explored is nitrogen source. To examine the effect of nitrogen source on selenite resistance, yeast were grown overnight in synthetic complete medium then inoculated into medium containing equimolar amounts of either allantoin, glutamate, ammonium, or urea with or without selenite and doubling times were measured (Figure 5).

As observed previously, selenite addition generally induced a small decrease in growth rate. All strains grew slowest in glutamate, and each strain grew fastest in a different medium, reflecting the genetic diversity of these strains. The ratio of the doubling time in medium with or without selenite was calculated for each strain under each nitrogen condition, and this was dubbed the S3/S0 ratio. The S3/S0 ratio is a proxy for selenite resistance with higher resistance indicated by a higher ratio and lower resistance indicated by a lower ratio. The S3/S0 ratios for all strains under each nitrogen condition were correlated with the amount of selenium accumulated for each strain when grown in OSAM (Table 3). Note, selenium accumulation was not assayed in the medium with varying nitrogen sources.

The only significant correlation between selenium accumulation and selenite resistance in a nitrogen source was in glutamate (*p*-value = 0.017). Thus, the result suggests that growth in glutamate affects selenite resistance in a manner proportional to selenium accumulation in OSAM.

### 3.2. Selection for Increased Selenite Resistance 

**Figure 6:**
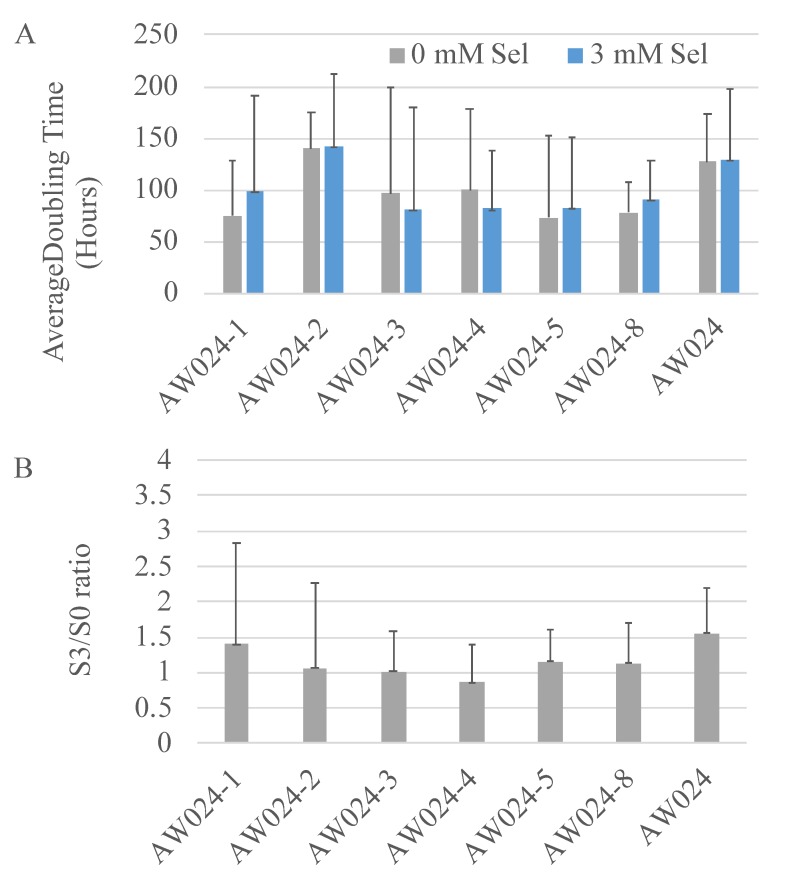
Growth rate of yeast mutants. (**A**) Strains were grown overnight in synthetic complete medium then inoculated into rich medium with 3% glycerol with 0 or 3 mmol L^−1^ selenite. The growth rate was monitored for 48 h, and maximal growth rate (doubling time) was determined as described in methods, and is shown on the graph, with the error bars representing the standard deviation of the measurements. (**B**) The S3/S0 ratio was determined by dividing the doubling time of the strain in 3 mmol L^−1^ selenite by the doubling time of the strain in 0 mmol L^−1^ selenite.

Based on the above analyses, the most significant correlation with a strain’s selenium accumulation was with that strain’s resistance to selenite when grown in glycerol. We hypothesized that cells selected for greater resistance to selenite when grown in glycerol may accumulate higher amounts of selenium. To test this, a culture of strain AW024, a haploid, auxotrophic derivative of strain EC1118, was randomly mutagenized with UV, then grown in rich medium with glycerol and 3, 5, then 10 mmol L^−1^ selenite. The few colonies that survived were separately recovered and grown to saturation, and the process was repeated. After three rounds of selection, six isolates were tested for their growth rate in medium containing glycerol with or without selenite, and compared with the parental strains (Figure 6A). The doubling time of strains grown in 3 mmol L^−1^ selenite were divided by the doubling time of strains grown without selenite to obtain a S3/S0 ratio (Figure 6B). A higher S3/S0 ratio indicates lower resistance to selenite.

**Figure 7:**
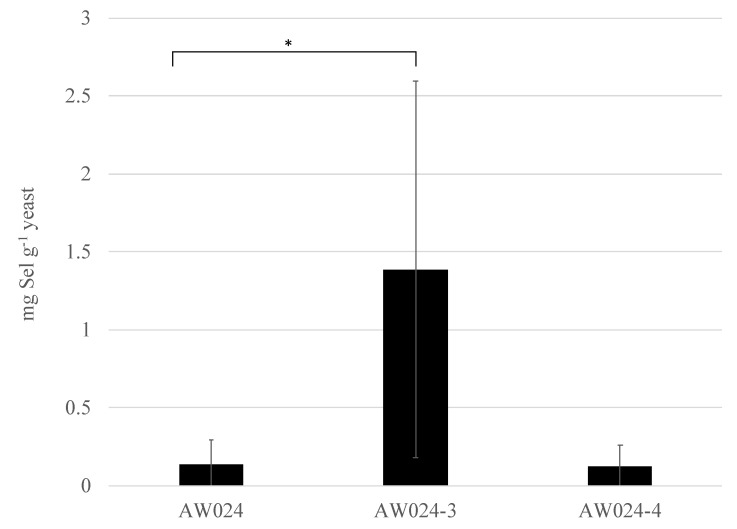
Selenium accumulation in yeast mutants. Yeast grew in synthetic complete medium overnight, then in optimized selenium accumulation medium in a shaking water bath set to 30 °C for 3 days, after which cells were washed three times in deionized water to remove extracellular selenite. Cells were spun down and the supernatant was discarded; wet cells were weighed, digested in nitric acid, and then the resulting solution was assayed for the quantity of ^82^Se using inductively coupled plasma mass spectrometry. Wet weight was converted to dry weight. Bar with asterisk indicates a significant difference (*p* < 0.05).

Strains AW024-3 and AW024-4 were identified as having the lowest S3/S0 ratios. These strains were next analyzed for selenium accumulation by growing strains in OSAM, and then assessing selenium accumulation (Figure 7).

The original parental strain AW024 and the isolate AW024-4 exhibited similar levels of selenium accumulation (135 and 125 ng Se g^−1^ yeast, respectively). However, the isolate AW024-3 exhibited approximately 10-fold greater levels of selenium accumulation than the parental strain AW024, a significant difference (1380 ng Se g^−1^ yeast; 1-tailed *t*-test *p* < 0.05). Thus we have identified a strain selected for selenite resistance in rich medium with glycerol as the sole carbon source that accumulates high levels of the metalloid when grown in OSAM.

## 4. Discussion

The goal of this study was to identify media conditions amenable to selection for yeast strains with increased selenium accumulation capability. *A priori*, one may expect that high selenium accumulation would correlate with high selenium resistance under all conditions. That is to say, that any strain with high selenium accumulation capability, would likely show high selenite resistance while growing in any medium as compared with strains that accumulate less selenium. We have seen that this is not the case. In fact, in most of the conditions tested, selenite resistance as measured by the ratio of the maximal growth rate in medium containing selenite to the maximal growth rate in medium lacking selenite, did not correlate with selenium accumulation. This is an interesting observation that is likely explained by media-dependent changes in selenite uptake, reduction, adsorption to the cell wall, toxicity, and/or intracellular reduction.

There was no clear correlation between the amounts of phosphorus, and most nitrogen and carbon sources and selenium accumulation in OSAM. However, a correlation was observed between strain-specific selenite resistance and accumulation when yeast were grown in synthetic medium containing glycerol or glutamate. Previous studies indicate that a selenite- and glycerol- containing medium severely retards yeast cell growth, but that the selenium is imported and transformed to L-selenomethionine [7]. The carbon source has also been shown to regulate the expression of Jen1, a lactate/selenite transporter and to cause changes in selenite tolerance [8,9]. As stated previously, the factors that determine selenium accumulation in OSAM or selenite resistance can be influenced by the rates of selenite uptake, reduction, adsorption to the cell wall, and toxicity, all of which can be modulated by growth medium [10]. Thus, we speculate that the rate of growth in glycerol- or glutamate- containing media in response to selenite is mainly affected by the factors that also affect selenium accumulation in OSAM. Namely, they would primarily be determined by the uptake rate and reductive capacity, and less so by adsorption to the cell wall. 

In the case of the correlation between selenite resistance in glutamate and selenium accumulation in OSAM, this may be due to the fact that glutamate is one of the three amino acids comprising glutathione. Thus its incorporation into glutathione to reduce selenite and its use as a nitrogen source for growth are both controlled by uptake rate, and perhaps other factors.

In addition to identifying the glycerol and glutamate effects, we selected six independent selenite-resistant yeast strains. One isolate exhibited 10-fold greater selenium accumulation in OSAM than the parental strain. The mechanism of increased accumulation in this strain is presently unknown, but we can propose several potential ways. Possibilities include mutation in the glutathione reductase gene *GLR1* or the selenodiglutathione vacuolar transporter *YCF1.* There may also be an as yet unidentified modifier of selenium accumulation. Further investigation of the genetics of accumulation is necessary to resolve this question.

## 5. Conclusions

The method described herein can be used to select for yeast with increased selenium accumulation. This method is doubly useful in that it does not require construction of GMO yeast, and as such would be suitable for use on yeast that would subsequently be sold in food products in the US and abroad.

## Figures and Tables

**Table 1:** Strains used in this study.

Name	Genotype	Source
EC1118	MATa/MATα wild-type	Lalvin
AW063	MATα *∆ade1 ∆pha2 ∆arg8 ∆lys2 ∆gal7*	This study
AW106	MATα *∆gal2 ∆mal2*	This study
Lalmin^®^ SE2000	wild-type	Lallemand Co.
AW024	MATα *ura*	This study
AW024-1	MATα *ura*	This study
AW024-2	MATα *ura*	This study
AW024-3	MATα *ura*	This study
AW024-4	MATα *ura*	This study
AW024-5	MATα *ura*	This study
AW024-8	MATα *ura*	This study

**Table 2:** Selenite resistance dependence on carbon source.

Carbon	Strain	Slope ^1^	Selenium Accumulation ^2^	*r*-Value ^3^	*t*-Value	*p*-Value
Ethanol	AW063	2033	0.95	–0.66	1.23	0.34
	AW106	2990	3.45			
	EC1118	894	3.10			
	Lalmin	−206	6.32			
Galactose	AW063	765	0.95	–0.67	1.29	0.33
	AW106	140	3.45			
	EC1118	1391	3.10			
	Lalmin	−318	6.32			
Glucose	AW063	718	0.95	–0.80	1.87	0.20
	AW106	99	3.45			
	EC1118	69	3.10			
	Lalmin	35	6.32			
Glycerol	AW063	1421	0.95	–0.99	8.69	0.013
	AW106	668	3.45			
	EC1118	932	3.10			
	Lalmin	152	6.32			
Lactate	AW063	1400	0.95	–0.64	1.19	0.36
	AW106	2191	3.45			
	EC1118	2558	3.10			
	Lalmin	−427	6.32			

^1^ The Slope column indicates the slope of the linear regression line fitted to the doubling time (Y-axis) correlated with selenite concentration (X-axis) for each strain under each carbon condition. ^2^ The selenium accumulation column refers to mg selenium per gram yeast when grown in OSAM (same data as in Figure 1). ^3^ The *r*-value for the correlation between the slope and the selenium accumulation in OSAM was calculated, and from this a *t*- and *p*-value were calculated as described in methods.

**Table 3:** Selenite resistance dependence on nitrogen source.

Nitrogen	Strain	S3/S0 ^1^	Selenium Accumulation ^2^	*r*-Value ^3^	*t*-Value	*p*-Value
Allantoin	AW063	1.44	0.95	0.62	1.11	0.38
	AW106	1.77	3.45			
	EC1118	1.08	3.10			
	Lalmin	0.79	6.32			
Glutamate	AW063	1.70	0.95	0.98	7.63	0.017
	AW106	1.19	3.45			
	EC1118	1.38	3.10			
	Lalmin	0.88	6.32			
Ammonium	AW063	1.27	0.95	0.31	0.46	0.69
	AW106	1.54	3.45			
	EC1118	1.55	3.10			
	Lalmin	1.40	6.32			
Urea	AW063	1.39	0.95	0.18	0.25	0.82
	AW106	1.08	3.45			
	EC1118	0.14	3.10			
	Lalmin	1.01	6.32			

^1^ The ratio of the growth rate of the indicated strain in medium without (SO) and with (S3) 3mmol L^−1^ selenite is indicated in the S3/S0 column. ^2^ The selenium accumulation column refers to mg selenium per gram yeast when grown in OSAM as depicted in Figure 1. ^3^ The *r*-value is the correlation between the value for S3/S0 for each strain in the indicated nitrogen source and selenium accumulation in OSAM.
